# Development and characterization of SiC nanofiber and hybrid reinforced composites for dental restorations

**DOI:** 10.1038/s41598-025-32575-w

**Published:** 2026-01-06

**Authors:** Samar E. Salem, Abdallah Shokry, A. H. Badran, W. Y. Ali, Ameer Ali Kamel

**Affiliations:** 1 Department of Mechanical Engineering, Faculty of Engineering, Fayoum University, Fayoum, 63514 Egypt; 2 Faculty of Engineering, Minia University, El-Minia, 61111 Egypt

**Keywords:** Dental composite materials, SiC nano fiber, Tribology, Mechanical properties, Morphology study, Thermal analysis, Engineering, Materials science, Nanoscience and technology

## Abstract

Enhancing the mechanical reliability of dental restorative materials is essential for improving long-term clinical performance. This study examined the mechanical, tribological, morphological, and thermal properties of Bis-GMA/TEGDMA (50/50 wt%) composites reinforced with silicon carbide (SiC) nanofibers and nanoparticles. Seven formulations were prepared: a control, three nanofiber composites (0.1–0.3 wt%), and three nanohybrid systems. All samples were photo-cured using strong, flashing, and gradually strong LED modes. Mechanical behavior was evaluated via Shore hardness and compression testing, while tribological performance was assessed using pin-on-disc wear analysis. SEM, XRD, and DSC provided structural and thermal characterization. SiC incorporation produced clear composition-dependent effects. Hardness increased by 3.5% in the 0.2% nanofiber composite relative to the control. The same formulation showed the greatest mechanical enhancement, with a 13.2% increase in compression strength, whereas the 0.3 wt% hybrid composite exhibited a 34% decrease, indicating overloading effects at higher hybrid content. Tribologically, both the 0.2% nanofiber and 0.3% hybrid composites demonstrated improved resistance to wear, exhibiting minimal weight loss. Curing mode significantly influenced all measured properties, with strong-mode curing yielding the highest overall performance. These findings highlight the potential and limitations of SiC-based reinforcement strategies for developing next-generation dental composites.

## Introduction

Dental restorative composites are commonly utilized in modern dentistry because of their cosmetic appeal, simplicity of implantation, and ability to attach to tooth structure. Their clinical effectiveness is frequently limited by mechanical failure, wear, and degradation in the oral environment^1,2^. This environment is challenging due to temperature fluctuations, mechanical stresses, frictional forces, and microbial activity. Together, these factors complicate the long-term performance and durability of restorative composites^3^. Advanced restorative systems, developed through advancements in nanomaterial research, are designed to improve dental caries treatment outcomes^4–7^. To endure the demanding conditions of the oral cavity, the major goal is still to achieve long-term durability combined with improved mechanical qualities and tribological performance^8^.

Nanocomposites consist of polymer matrices that are reinforced with nanoscale fillers, including fibers, whiskers, platelets, or nanoparticles. These materials provide a high interfacial surface area, facilitate enhanced load transfer, and exhibit improved mechanical and tribological properties^9,10^. As it directly affects mechanical qualities, tribological traits, durability, and biocompatibility, the matrix composition of dental restorative materials is a crucial factor in determining their overall performance^11^. The kinds of monomers used, their ideal concentration ratios, the polymerization procedure, and the curing technique used all have a substantial impact on its composition^12^. To obtain the required material properties, the choice of polymers and their exact ratios must be carefully tuned. The polymerization procedure and curing methods (such as light-, chemical-, or dual-curing) are essential for guaranteeing the restorative material’s appropriate cross-linking, structural integrity, and long-term stability^13^. In dental restorative materials, Bis-GMA (Bisphenol A-glycidyl methacrylate) serves as a primary dental resin material and is frequently utilized alongside TEGDMA (Triethylene glycol dimethacrylate) and UDMA (Urethane dimethacrylate), serving as a diluent to reduce viscosity^14^. This families make up the most well-known and thoroughly researched combination in numerous dental applications^15–17^. This system is highly regarded for its balanced mechanical properties, polymerization efficiency, and clinical performance^18–21^. Dental restorative materials frequently include additives in addition to the main matrix monomers, which are essential for promoting and enhancing the polymerization process^22^. In light-cured systems, photoinitiators and co-initiators are particularly essential among these additives. Camphorquinone (CQ), the most widely used photoinitiator, absorbs blue light (around 468 nm) and produces free radicals to start the polymerization of methacrylate-based resins^23^. Usually, amine compounds such as EDMAB (Ethyl 4-dimethylaminobenzoate) and DMAEMA (Dimethylaminoethyl methacrylate) are used as co-initiators to enhance photoinitiators systems, increasing the degree of monomer conversion and the efficiency of free radical generation^24^.

Fillers are essential components in current dental restorative materials because they improve the composite’s mechanical strength, wear resistance, polymerization shrinkage control, and cosmetic features^18,21,25,26^. Filler size, morphology, and concentration markedly impact the properties of restorative composites, and nanoscale fillers, in particular, are broadly employed in modern dental materials^27–29^.

Hybrid nanocomposites combining different filler types can provide synergistic improvements in hardness, compressive strength, and wear resistance^9,30–32^. Recent studies on hybrid dental composites have demonstrated clear improvements in wear resistance, frictional performance, and thermo-mechanical stability with the incorporation of micro- and nano-scale particulates, as confirmed by comparative evaluations on hybrid systems^33,34^. Moreover, ceramic-based reinforcements - such as alumina–zirconia particulates and tri-calcium phosphate/silica systems have shown notable enhancements in strength and overall performance, emphasizing the effectiveness of tailored inorganic fillers in dental resin matrices^35,36^. Furthermore, broader reviews highlight that filler size, morphology, and distribution critically influence the physical, mechanical, and tribological behavior of reinforced polymer composites, supporting ongoing advancements in dental material optimization^37,38^. In addition to these findings, recent literature highlights the wide range of factors influencing the performance of dental composite materials.

Comprehensive reviews emphasize the need to understand physical, chemical, mechanical, thermal, tribological, and biological behaviors when developing next-generation restorative systems^39^. Experimental investigations have further shown that inorganic fillers, such as aluminum oxide, titanium oxide, hydroxyapatite, tricalcium phosphate, and zirconia can substantially enhance physico-mechanical strength, thermal stability, and tribological performance, confirming the effectiveness of tailored ceramic reinforcements in advanced composite formulations^40,41^.

Silicon carbide (SiC) is a highly effective ceramic filler known for its hardness, chemical stability, and thermal resistance. Its integration into dental composites, as nanofibers or nanoparticles, has been reported to enhance mechanical strength, wear resistance, and thermal stability, while maintaining compatibility with polymeric resin matrices^1,2,10,30^. Despite these advantages, excessive filler loading or hybrid combinations can adversely affect mechanical properties, highlighting the need for systematic evaluation^9^.

Although MCDM (Multi-Criteria Decision-Making) techniques have gained increasing attention for ranking and selecting dental restorative composite materials, several limitations should still be considered. First, many methods depend on subjective expert judgement, which may introduce bias unless supported by objective weighting techniques, as shown in entropy–VIKOR approaches^42,43^. Second, the final ranking is highly method-dependent, with different algorithms—such as the R-method^44^, entropy-VIKOR^42,43^, FAHP–TOPSIS and FAHP–FTOPSIS^45,46^, ceramic composite optimization methods^47^, and hybrid AHP–TOPSIS frameworks^48^—often yielding different outcomes for the same dataset. Third, the treatment of uncertainty remains limited, as most studies do not address measurement variability or error propagation. Fourth, correlations among key performance attributes (e.g., hardness, wear, friction, thermal properties) may affect the reliability of weighting schemes. Finally, sensitivity analysis and generalizability to clinical conditions are still insufficient in many investigations. These considerations highlight the need for more robust and comprehensive decision-making frameworks when evaluating dental restorative composites^42–48^.

The novelty of this study lies in the systematic evaluation of Bis-GMA/TEGDMA dental composites reinforced with silicon carbide (SiC) in the form of nanofiber and hybrid composites, under different photo-curing modes. SiC was selected due to its high hardness, thermal stability, chemical inertness, and compatibility with polymer matrices, which make it a promising filler to enhance the structural and functional performance of dental composites. Bis-GMA/TEGDMA (50/50 wt%) was chosen as the matrix because of its widespread use in restorative dentistry and well-characterized mechanical properties.

The objectives of this study are to evaluate the effect of SiC nanofibers and their hybrid combinations on the hardness and compression strength of dental composites, to assess the wear resistance of these composites under different curing modes, and to analyze their morphological, structural, and thermal properties. In addition, the study aims to determine the optimal filler type and content required to enhance the mechanical and tribological performance of the developed composites.

## Materials and methods

### Materials used

**Fig. 1:**
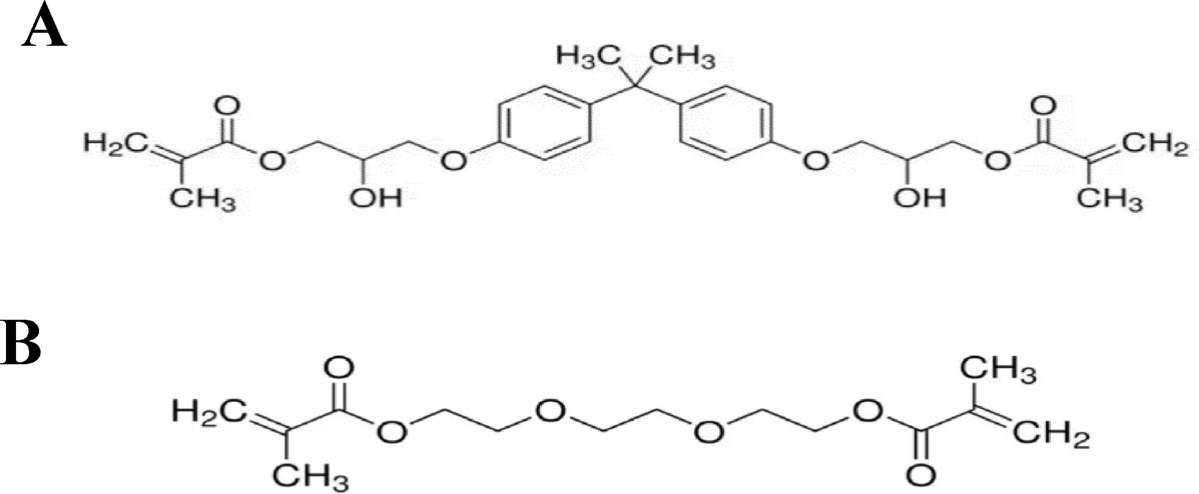
The chemical structures of matrix components: **A** Bis-GMA, **B** TEGDMA.

Different compositions of dental composites were fabricated. The dental resin consisted of a base matrix, Bis-GMA (C_29_H_36_O_8_), and a diluent, TEGDMA (C_14_H_22_O_6_). The photoinitiator system comprised camphorquinone (CQ) as the initiator and DMAEMA as the co-initiator. Fillers included silicon carbide (SiC) nanofibers (diameter < 2.5 μm, aspect ratio ≥ 20) and 59 nm SiC nanoparticles. γ-Methacryloxypropyl trimethoxysilane (γ-MPS) was used as a silane coupling agent. All materials are obtained from Sigma-Aldrich Company, Germany. The chemical structures of matrix components are illustrated in Fig. 1.

### Silanization process

To improve adhesion with the dental resin matrix, SiC nanoparticles and nanofibers underwent surface modification using γ-methacryloxypropyl trimethoxysilane (γ-MPTS). The silanization solution was prepared by combining ethanol and distilled water (70:30, v/v), adjusting the pH to 4.2 ± 0.1 with acetic acid. The fillers were dispersed in this solution, stirred briefly at room temperature, and subsequently kept under reflux at 70 °C for 4 h following a 1 h immersion period. After treatment, the materials were thoroughly rinsed with ethanol and oven-dried at approximately 100 °C for 24 hr^49,50^.

### Samples preparation

**Fig. 2:**
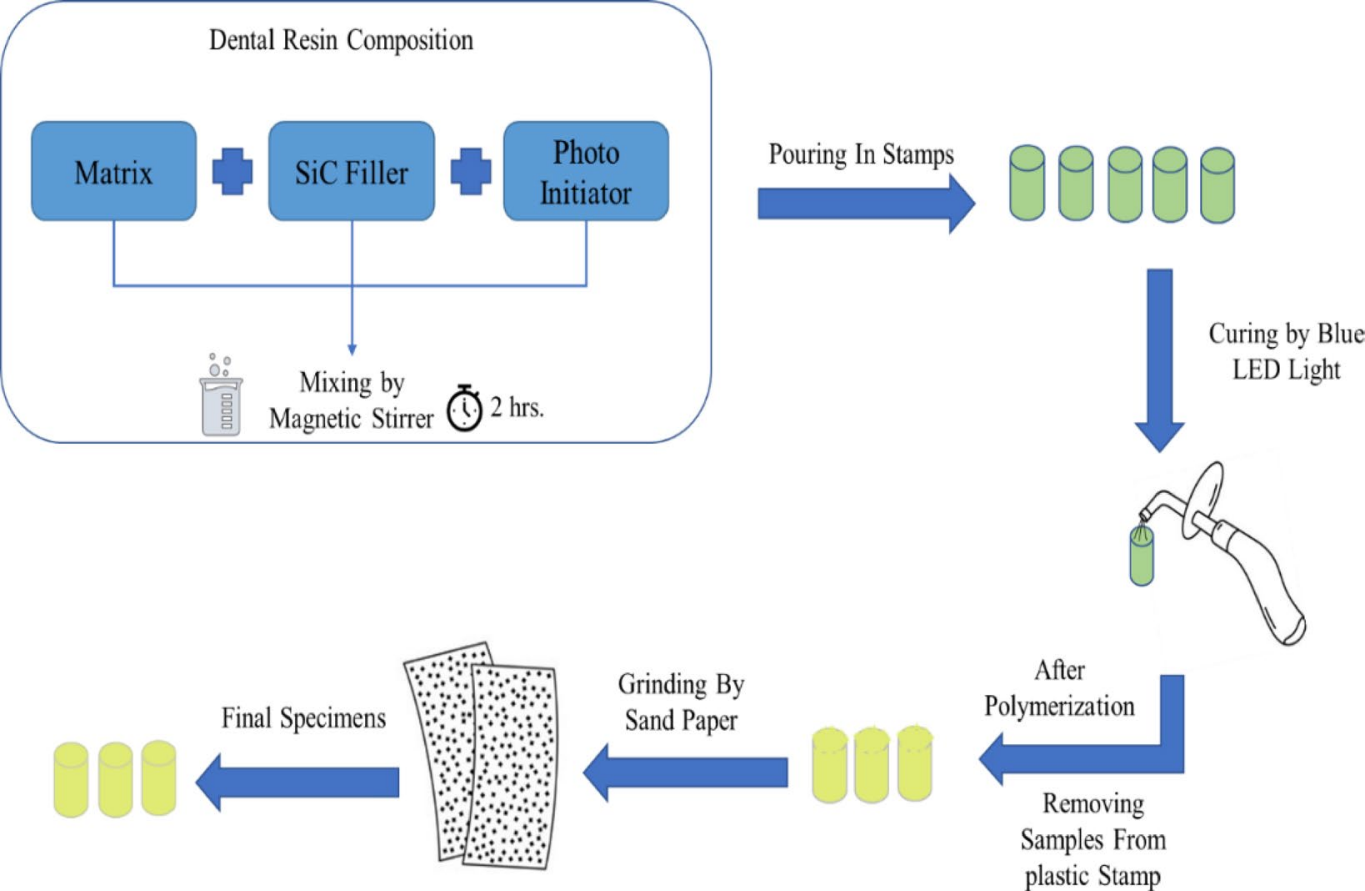
Steps of preparation process.

**Fig. 3:**
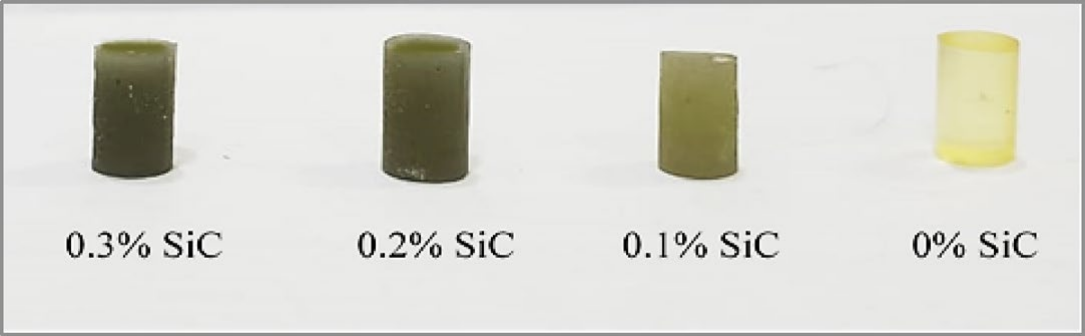
Final specimens.

A total of approximately 150 specimens were fabricated for the experimental study. Seven composite groups were designed: a control (0 wt% SiC), three nanofiber composites (0.1, 0.2, 0.3 wt%), and three nanohybrid composites containing equal proportions of nanofibers and nanoparticles at the same weight fractions. The resin matrix consisted of 49.5% Bis-GMA, 49.5% TEGDMA, 0.7% DMAEMA, and 0.3% CQ, mixed magnetically at 200 rpm for 2 h under dark conditions, with SiC fillers incorporated after 1 h. The mixtures were cast into cylindrical molds (6 mm × 10 mm) and polymerized with a blue LED curing unit (420–480 nm, 1200–2000 mW/cm²) for 60 s per side under three curing modes: strong, flashing, and gradually strong. After curing, specimens were demolded and polished using 2000-grit sandpaper. Figure 2 illustrates the preparation process. Figure 3 presents the cured specimens, showing the effect of filler content on color variation, ranging from light to dark.

## Experimental tests

### Hardness test

The hardness of the composites was measured using a Shore D durometer in compliance with ASTM D-2240^51^. Each specimen was positioned on a rigid, level surface, after which the indenter was applied vertically to achieve full contact between the specimen and the surface. For each of the 21 conditions investigated, three specimens were tested, and 10 measurements (five from the top and five from the bottom surface) were recorded per specimen.

### Friction and wear test

A pin-on-disk apparatus was employed to investigate the wear behavior and coefficient of friction of the specimens. Cylindrical samples (6 mm diameter × 10 mm length) were mounted in a chuck oriented perpendicularly to a flat PMMA disk. The tests were carried out under dry conditions at a constant sliding speed of 75 rpm, with five different normal loads (4, 6, 8, 10, and 12 N) were applied via the lever arm to maintain contact between the specimen and the rotating disk. Each trial was conducted for 30 s. Wear behavior was evaluated based on weight loss measurements of the samples before and after the wear test using a digital balance with 0.0001 g precision. The coefficient of friction^52^ was determined using the standard equation:

$$\mu _{{\text{s}}} = {\text{ F}}_{{\text{s}}} /{\text{F}}_{{\text{n}}}$$

Where µ_s_ represents the coefficient of friction, F_s_ is the friction force, and F_n_ is the normal load. The data obtained from the friction and wear tests were analyzed using analysis of variance (ANOVA).

### Compression test

Compressive strength was measured using a Zwick Z010 testing machine (10 kN load cell, Germany) following ASTM D695 at a crosshead speed of 1.33 mm/min. Tests were conducted until specimen fail, with three replicates per condition, and mean values were reported.

## Morphology study

### X-Ray diffractometer

X-ray diffraction (XRD) analysis was employed to evaluate filler dispersion and crystallinity of the samples. Measurements were conducted over a diffraction angle range of 0° ≤ 2θ ≤ 80°using a radiation wavelength of 1.54060 nm. The analysis was performed at the CMRDI, in Egypt.

### Scanning electron microscope

Examination of the worn surface morphology was performed by SEM at the CMRDI. To improve surface conductivity and image resolution, the specimens were sputter-coated with a thin gold layer prior to analysis.

## Thermal analysis

### Differential scanning calorimetric (DSC)

Differential scanning calorimetry (DSC) was employed to monitor the glass transition temperature and relate filler content to cross-link density and polymer rigidity. Heat-flux DSC was carried out using two identical alumina crucibles, with ~ 20 mg of sample placed in one crucible and the other left empty as a reference. The analysis was performed in the temperature range of 50–600 °C at a heating rate of 10 °C/min. All measurements were conducted at the CMRDI.

## Results and discussions

### Hardness test

**Fig. 4:**
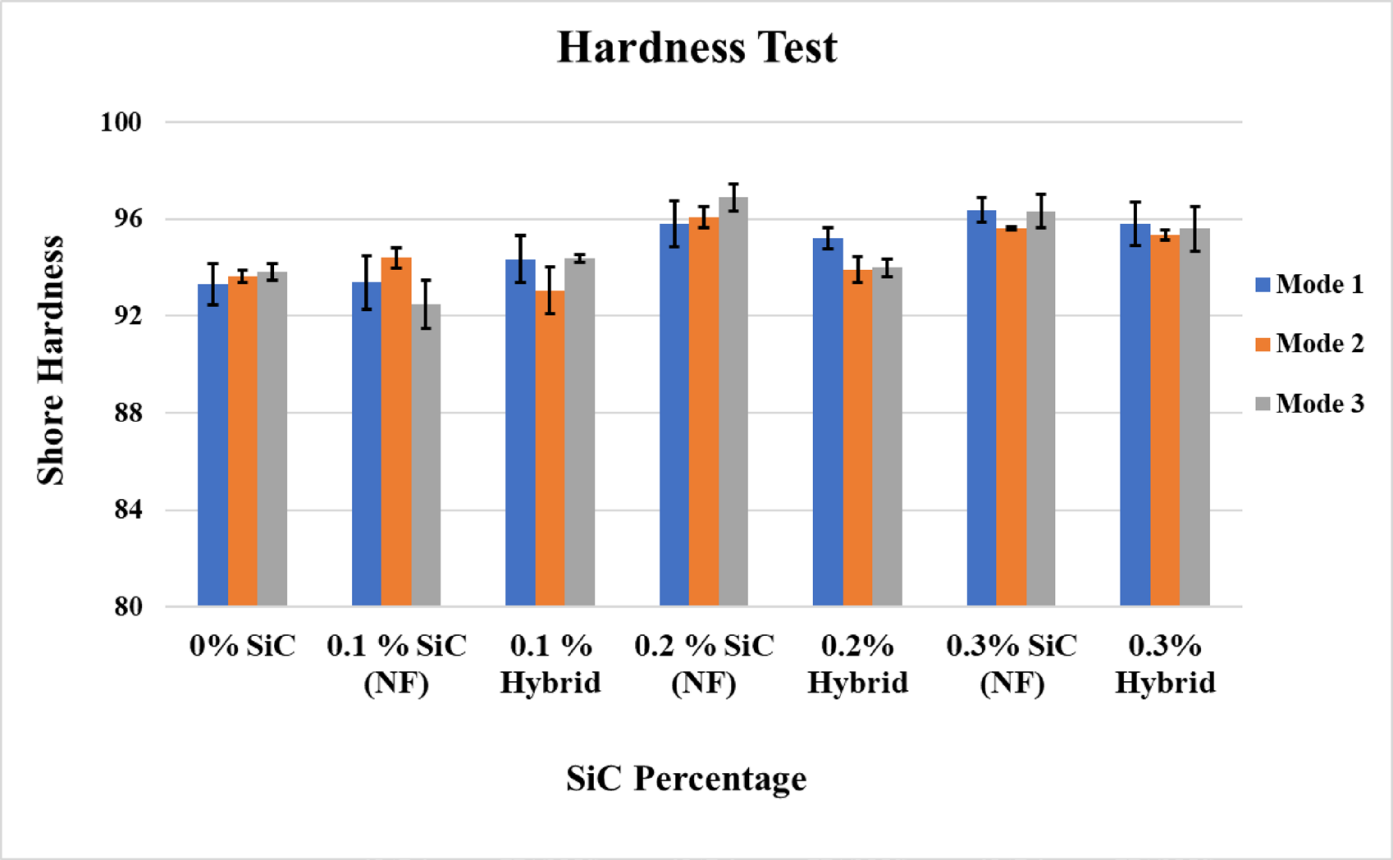
Hardness strength for nano fiber and nano hybrid composite.

Figure 4 illustrates the correlation between SiC nanofiber/nanoparticle content and hardness under the three curing modes. The maximum hardness was recorded at 0.3 wt% hybrid SiC cured under mode 2, showing an improvement of approximately 3.9% compared to the control. This was followed by the 0.2 wt% SiC nanofiber sample cured under mode 3, with a 3.5% increase relative to the control. The hardness test revealed that the incorporation of SiC enhanced the hardness strength of the dental composite, consistent with previous findings^25,53^. The incorporation of nanoparticles within nanofibers reduces interparticle spacing and strengthens bonding, while their high surface area enhances interfacial interactions with the resin matrix, thereby improving the overall properties of the composites, as supported by literature^54^.

### Friction and wear test

#### Coefficient of friction

**Fig. 5:**
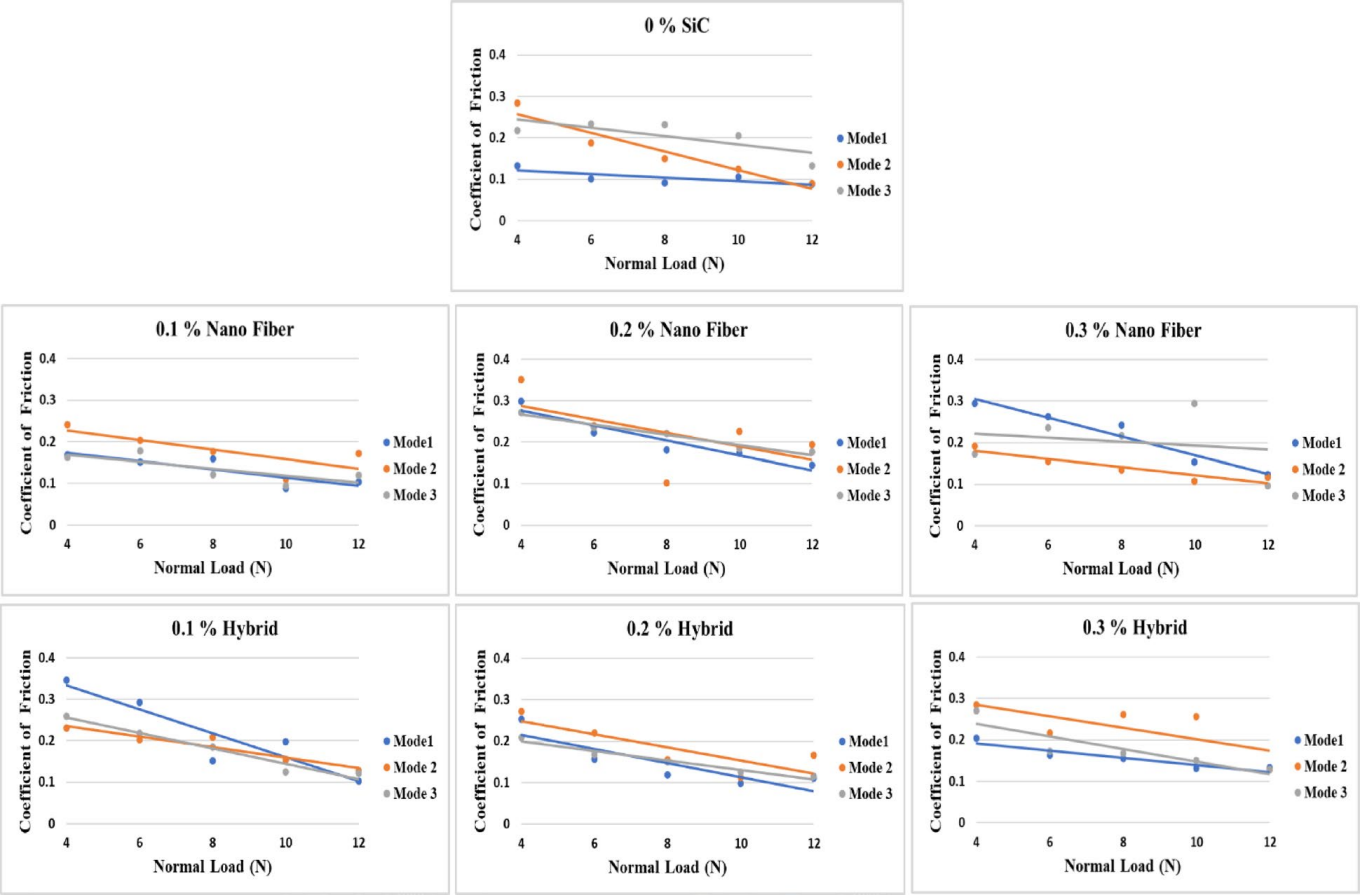
Coefficient of friction at different normal loads for nano fiber/ nano hybrid composite with different SiC content.

The coefficient of friction (COF) for nanofiber and nano-hybrid composites was determined for the three curing modes and under five normal loads (4, 6, 8, 10, and 12 N), as presented in Fig. 4. The results indicate that COF decreases as the applied normal load increases. This trend is attributed to friction-induced temperature rise, which facilitates the development of a stable tribolayer, thereby lowering interfacial resistance and reducing frictional forces. On the other hand, the incorporation of nanofibers and nanoparticles resulted in a noticeable increase in COF compared to the control group. This behavior can be attributed to the formation of micro-asperities on the composite surface, which promote mechanical interlocking with the counterface and thereby raise sliding resistance. In addition, the detachment or fragmentation of fibers and particles may generate third-body debris, contributing to abrasive or three-body wear mechanisms and further amplifying the friction response. Moreover, it is evident from Fig. 5 that samples cured under mode 1 exhibited the lowest coefficient of friction across the tested conditions, highlighting the influence of curing mode on tribological performance.

#### Weight loss

For further analysis, the wear-induced weight loss was quantified to determine which composite demonstrated the lowest degradation under testing conditions. While the coefficient of friction provides insight into interfacial interactions and sliding behavior, weight loss directly reflects the long-term durability of restorative dental materials. Therefore, this parameter was emphasized as a primary performance indicator, allowing comparison of composites in terms of both mechanical stability and clinical longevity.

**Fig. 6:**
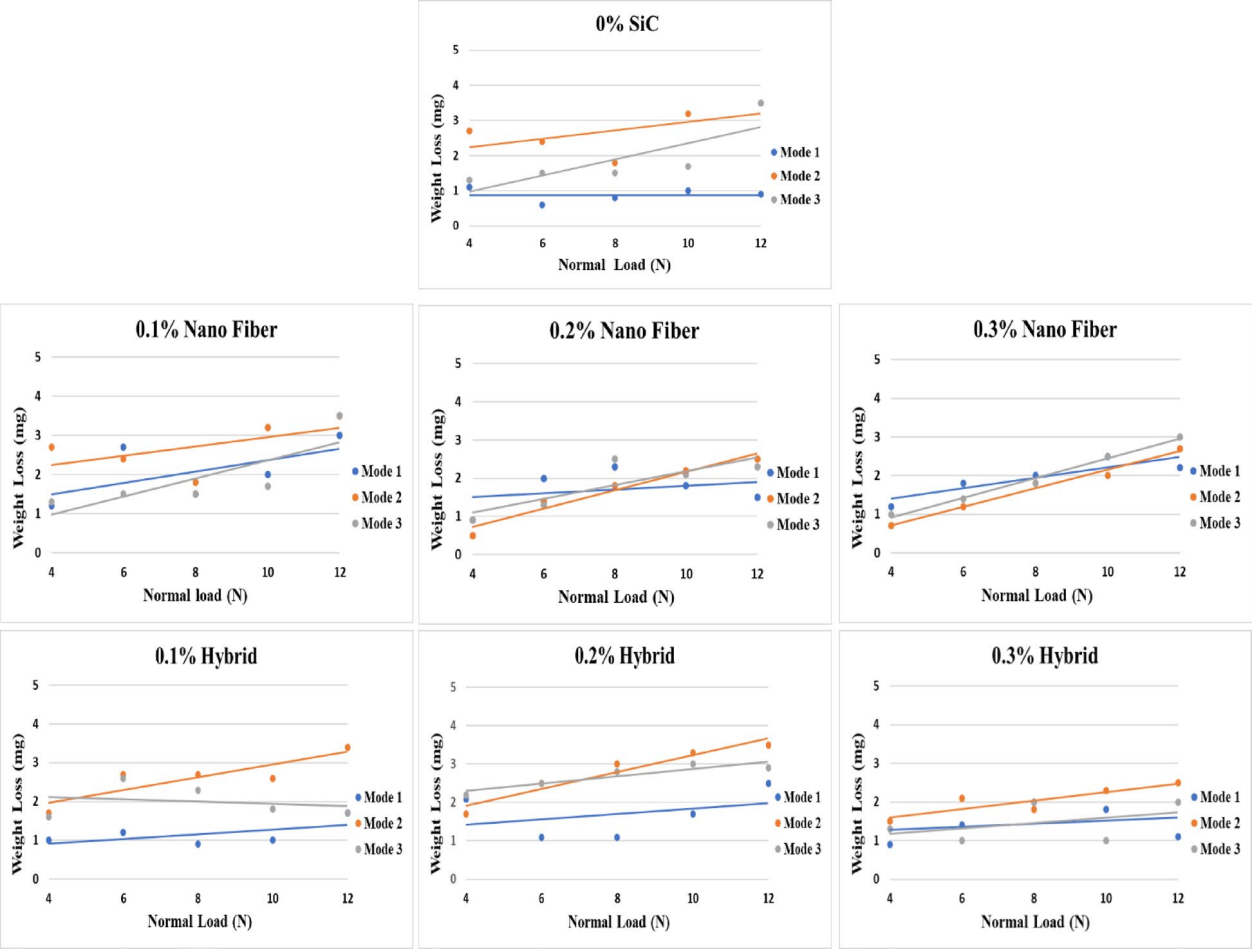
Weight loss of nano fiber and nano hybrid composites due to wear and friction test.

The weight loss of nanofiber and nanohybrid composites is presented in Fig. 6. The findings indicate that wear loss increases consistently with increasing normal load. Such a pattern arises from the elevated contact pressures at higher loads, which intensify abrasive and adhesive wear mechanisms despite the relatively low coefficient of friction. Under such conditions, surface interactions become more aggressive, leading to severe plastic deformation, micro-fracturing of the resin matrix, and eventual detachment of fillers from the composite surface, thereby accelerating material removal.

#### Analysis of variance for weight loss

ANOVA results for weight loss revealed that all investigated factors had a statistically significant influence, as evidenced by P-values less than 0.05 for each factor as indicated in Table 1.

**Table 1:** ANOVA analysis for weight loss due to friction against PMMA.

Source	DF	SS	MS	F	*P*
SiC%	6	6.458	1.0763	3.11	0.008
Mode	2	10.653	5.3264	15.38	0.000
Load (N)	4	15.591	3.8979	11.26	0.000
Error	92	31.860	0.3463		
Total	104	64.562			

**Fig. 7:**
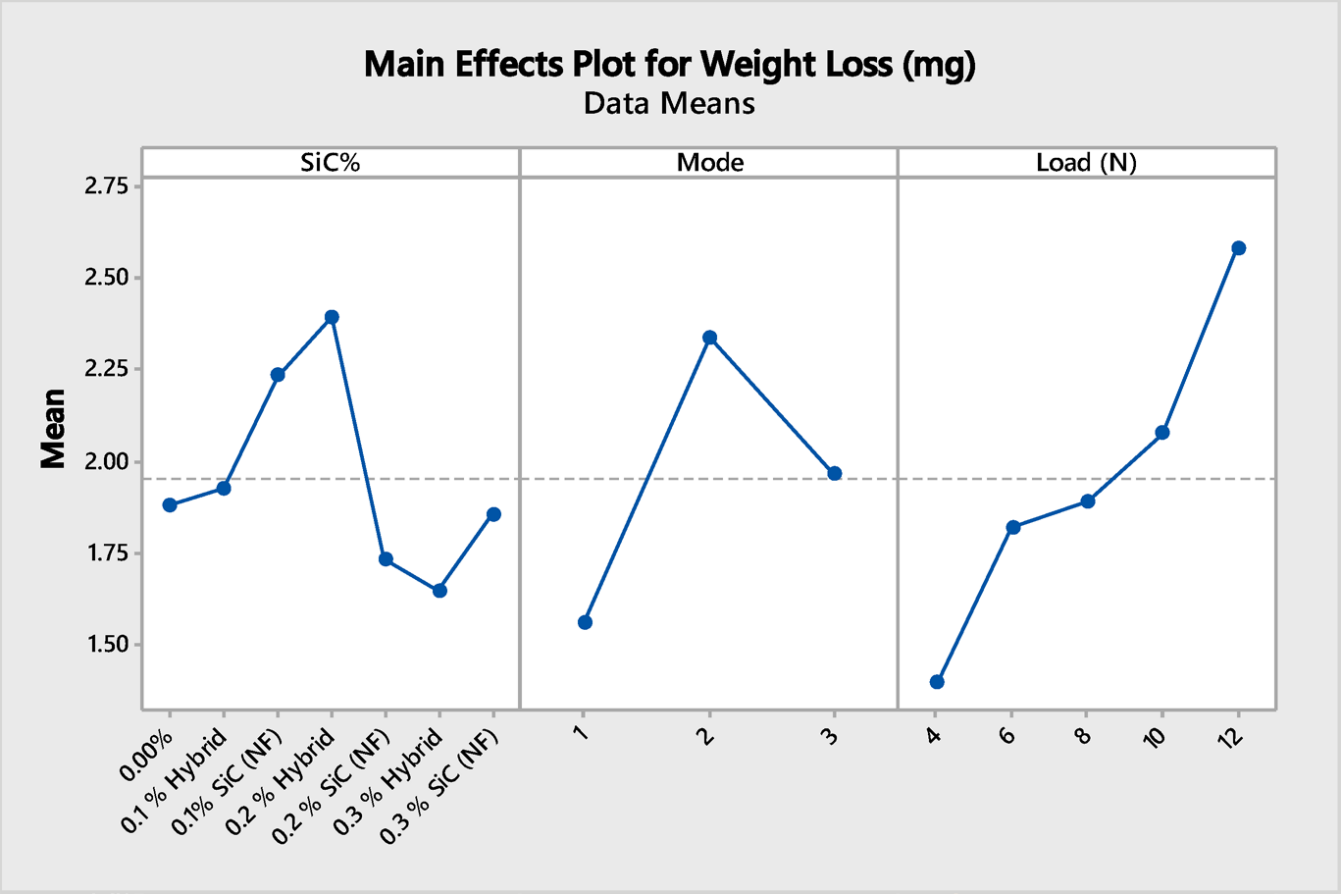
Main effect plot for weight loss.

As shown in the main effects plot (Fig. 7), the applied load had a strong influence on weight loss, with higher loads resulting in significantly greater wear. Also, increasing SiC content reduced weight loss, most notably in the 0.3% hybrid composites, followed by the 0.2% nanofiber composites. Curing mode also played a role, as samples cured under mode 1 exhibit the lowest wear.

**Fig. 8:**
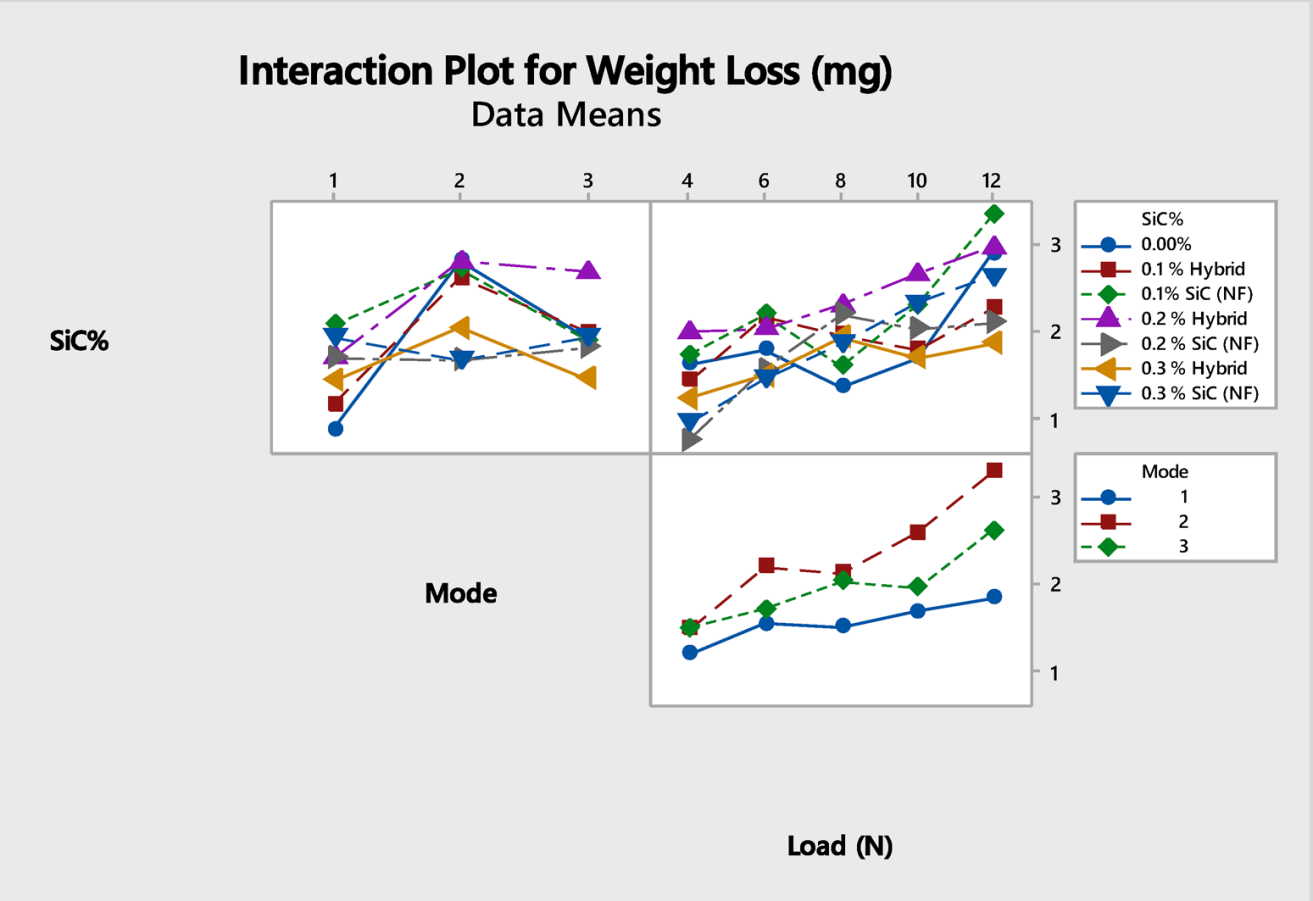
Interaction plot showing the effect of SiC content, load, and curing mode on weight loss.

The interaction plot in Fig. 8 illustrates that the influence of SiC content on weight loss is dependent on both applied load and curing mode. Minimal effects are observed at low loads, whereas at higher loads, the reinforcing role of SiC becomes more pronounced, indicating a notable interaction between filler content, load, and curing conditions.

### Compression test

**Fig. 9:**
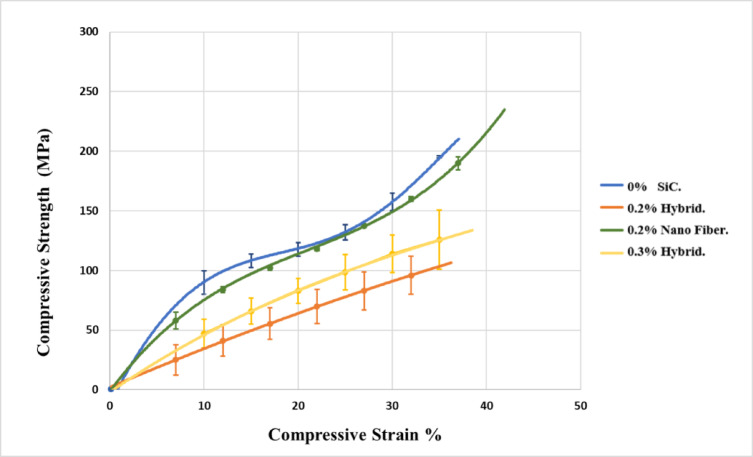
Compressive strength for 0%, 0.2% hybrid, 0.2% nano fiber, and 0.3% hybrid samples cured by mode 1.

Samples for compression test were selected based on ANOVA and Main Effects Analysis, focusing on the most influential factors. Figure 9 shows the variation in compressive strength under mode 1 curing for nanofiber and nanohybrid composites compared with the control sample. The 0.2% nanofiber-reinforced composite achieved the highest compressive strength, highlighting the reinforcing effect of nanofibers. In contrast, all nanohybrid composites exhibited lower compressive strength than the control group, indicating a reduced ability to resist compressive stress. This reduction can be attributed to the disruption of a continuous nanofiber network by the presence of nanoparticles, which weakens the structural integrity required to bear compressive loads^55^. In addition, a high filler content can restrict light penetration during curing, leading to insufficient polymerization, particularly in the deeper regions of the composite. The central zone, which receives minimal light exposure, consequently exhibits the lowest degree of conversion, making it the weakest region and less capable of resisting compressive stresses^56^.

The compressive strength of nanofiber-reinforced composites is higher than that of nanohybrid composites with the same SiC concentration of 0.2%. This is because nanofibers have a higher aspect ratio than nanoparticles, which results in larger length-to-diameter proportions and improves mechanical interlocking and the composite’s overall reinforcing^57,58^. Additionally, the dispersion and preferential orientation of nanofibers inside the matrix material guarantee that these fibers will efficiently bridge the fracture faces when microcracks caused by masticatory forces begin to form and spread. By preserving structural integrity throughout the fracture plane, this crack bridging mechanism promotes load transmission and raises the composite’s total fracture toughness^59^.

Compressive strength values, extracted from the stress–strain curve (Fig. 9), show that the control composite (0% SiC) reached 212.5 MPa. The 0.2% SiC nanofiber composite exhibited the highest strength at 240.6 MPa, confirming its reinforcing efficiency. In contrast, the 0.2% hybrid composite displayed a substantially reduced strength (102.8 MPa**)**, while the 0.3% hybrid composite achieved 139.3 MPa.

### Results of morphology study

#### X-Ray diffractometer

**Fig. 10:**
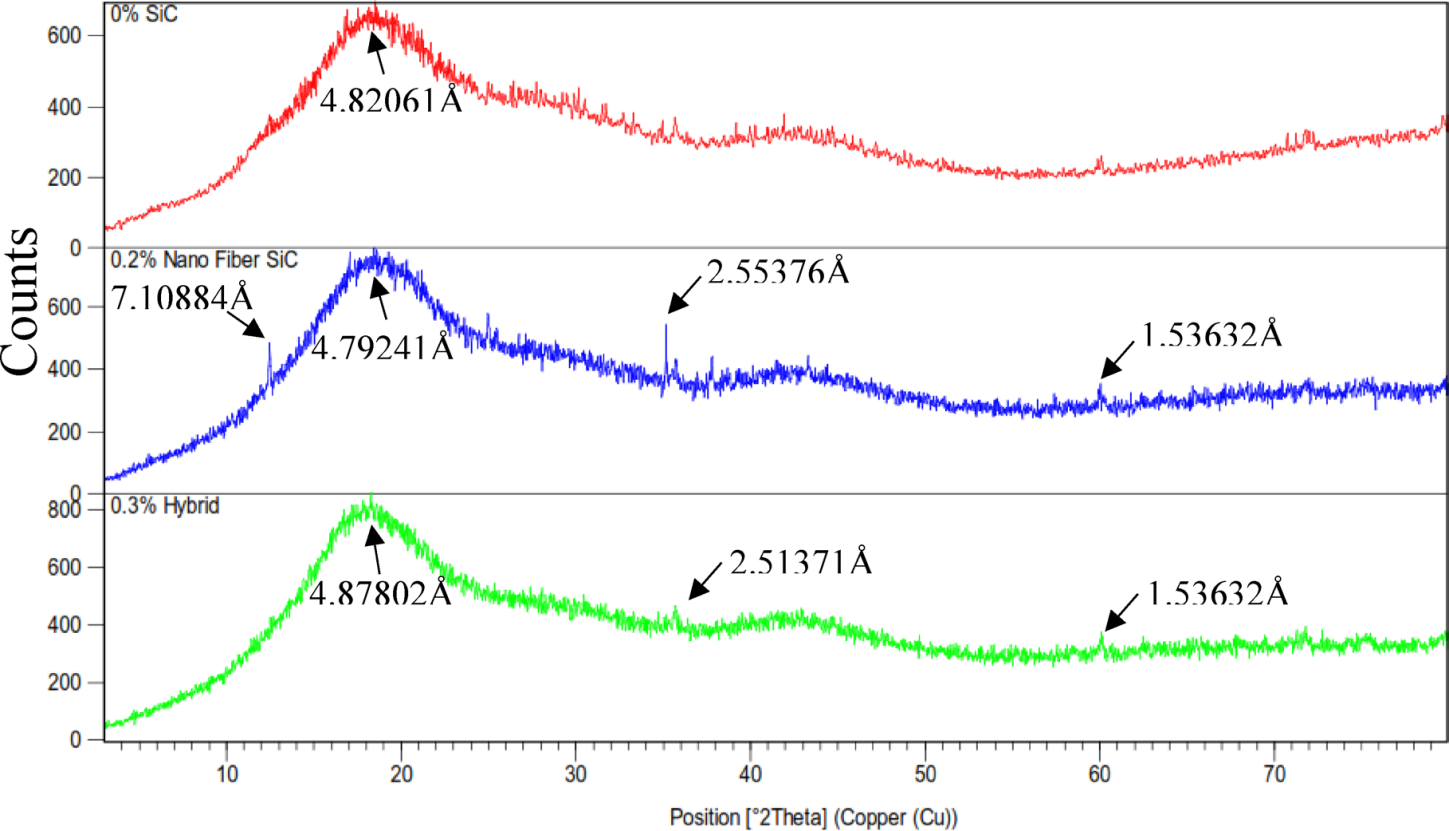
XRD Pattern for control sample, 0.2% nano fiber sample, and 0.3% hybrid sample cured by mode 1.

The structural properties of the nano fiber and nano hybrid dental composite systems were examined using X-ray diffraction (XRD) analysis. The control sample (0% SiC polymer composite), 0.2% nanofiber-reinforced composite, and 0.3% hybrid composite’s XRD patterns are shown in Fig. 10. All samples showed broad amorphous peaks typical of the Bis-GMA/TEGDMA matrix. The control sample (0% SiC) exhibited a fully amorphous pattern, while the 0.2% nanofiber composite displayed sharp diffraction peaks, confirming the incorporation of crystalline SiC and a shift toward semicrystalline behavior. A distinct peak at 2θ = 35.6° (d-spacing = 2.51 Å) was identified, corresponding to the (111) plane of cubic β-SiC (ICSD 28389)^60,61^. The diffraction peak at 2θ = 35.6° (d-spacing = 2.51 Å) confirms the incorporation of crystalline β-SiC nanofibers, matching the cubic lattice parameter (a = 4.36 Å) and indicating phase purity. Additional reflections, such as the (220) plane at 60.0°, reinforce this crystalline signature within the amorphous polymer matrix. The 0.3% hybrid composite displayed an intermediate pattern, suggesting more complex phase interactions.

#### Scanning electron microscope

**Fig. 11:**
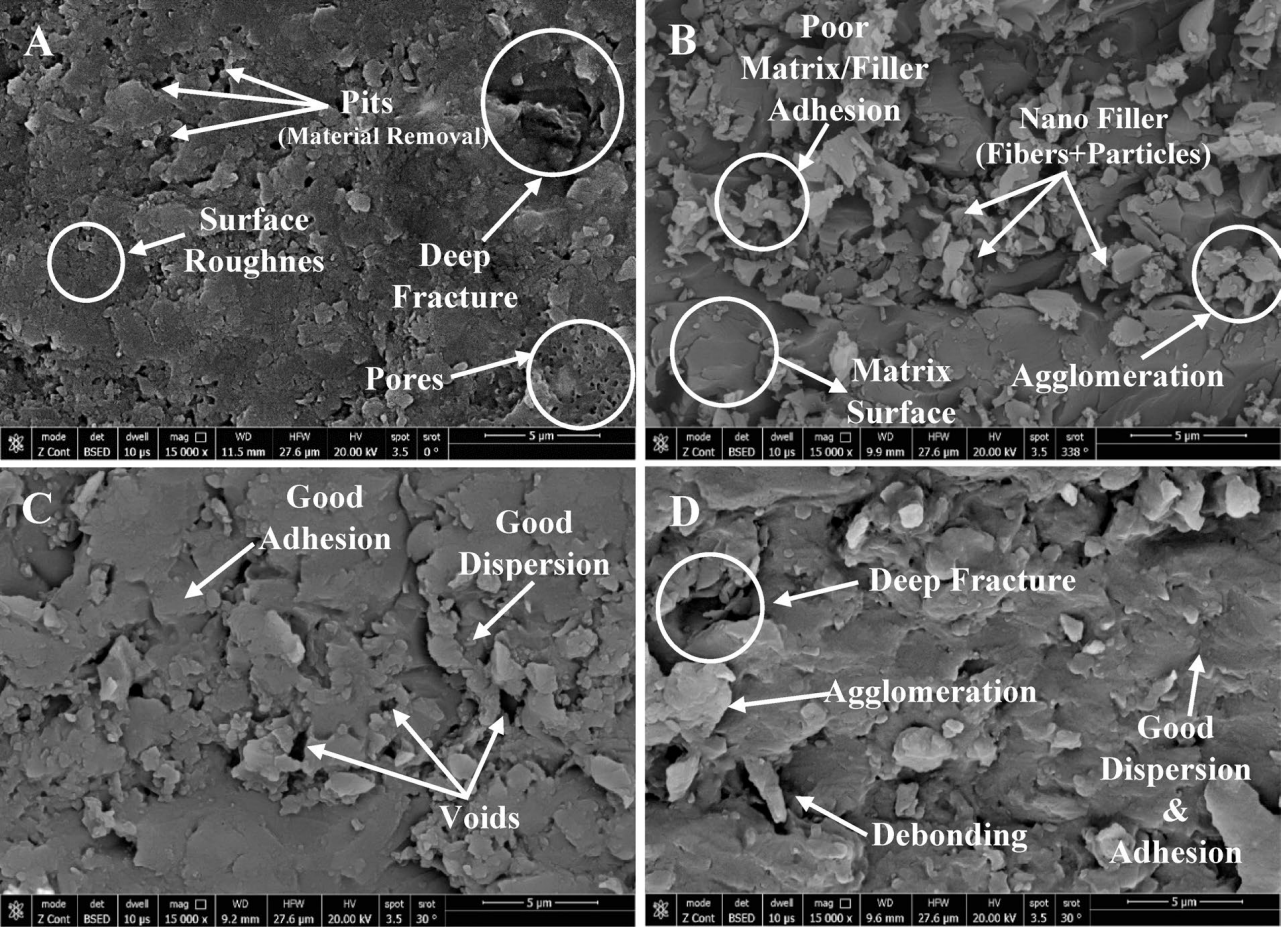
SEM images for samples cured by mode1: **A** Control sample 0% SiC, **B** 0.2% Hybrid, **C** 0.2% Nano fiber, **D** 0.3% hybrid.

Figure 11 presents SEM micrographs of the control sample, 0.2% hybrid, 0.2% nanofiber, and 0.3% hybrid composites. The control sample (Fig. 11A) shows an amorphous resin matrix with surface roughness, pores, and microfractures from curing and abrasive wear. In the 0.2% hybrid composite (Fig. 11B), sharp phase boundaries and localized agglomerates indicate poor filler–matrix adhesion and uneven dispersion, limiting morphological improvements. Agglomerations cause the mechanical strength of dental composite to decrease^62^. By contrast, the 0.2% nanofiber composite (Fig. 11C) exhibits smoother transitions, stronger interfacial bonding, and more uniform dispersion, though some microvoids remain. Increasing the hybrid filler to 0.3% (Fig. 11D) improves phase continuity and adhesion, but agglomeration and debonding persist at higher loadings, contributing to stress concentration and fracture formation.

**Fig. 12:**
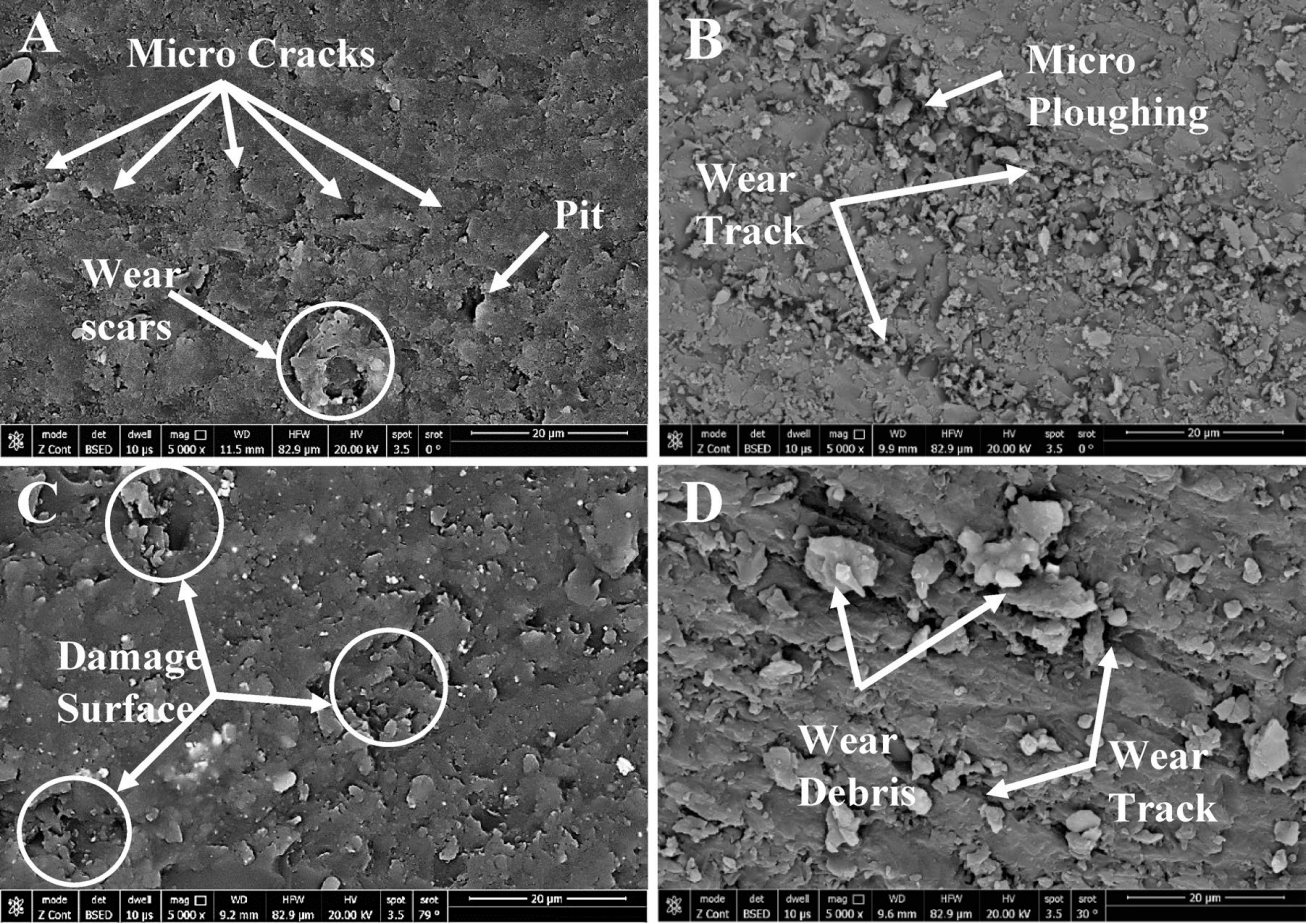
Wear surface morphology for samples **A** Control sample 0% SiC, **B** 0.2% Hybri, **C** 0.2% Nano fiber, **D** 0.3% hybrid.

The SEM micrographs indicating the worn surface morphology for the same samples are shown in Fig. 12. The mostly amorphous structure of the control sample (Fig. 12-A) is responsible for the visible wear scars, pits, and microcracks on its worn surface. The 0.2% hybrid composite (Fig. 12-B), on the other hand, shows a discernible improvement in surface morphology, with micro-ploughing evidence suggesting a more regulated wear mechanism.

The 0.2% nanofiber-reinforced sample, shown in Fig. 12-C, exhibits additional enhancements in fiber–matrix interfacial adhesion together with a decrease in surface imperfections. On the other hand, the 0.3% in the hybrid composite in Fig. 12-D indicates the appearance of wear debris on the surface, indicating a rise in material deterioration under wear circumstances.

### Thermal analysis

#### Differential scanning calorimetric (DSC)

**Fig. 13:**
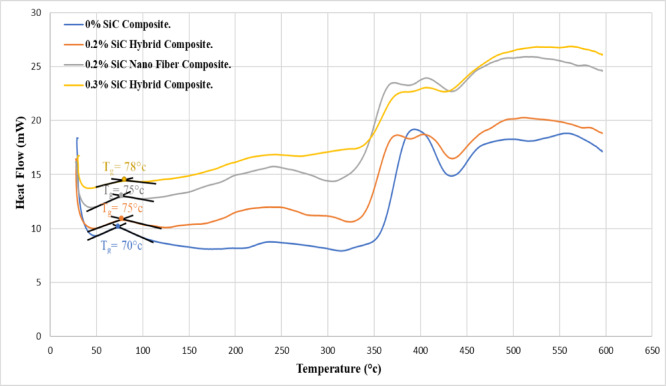
DSC Thermograms for The Four Conditions Samples Cured by Mode1 : 0% SiC, 0.2% Nano Fiber, 0.2% Nano Hybrid, and 0.3% Nano Hybrid Composites.

Figure 13 displays DSC thermograms of four selected samples based on ANOVA. All samples exhibited similar thermal responses, consistent with the amorphous nature of the dental composite, with glass transition temperatures (Tg) ranging from 70 °C to 78 °C. The control sample (0% SiC) recorded the lowest Tg (70 °C), while the 0.3% nano-hybrid composite reached the highest (78 °C). The control sample also showed a broad exothermic peak between 350 and 440 °C, indicating thermal degradation, whereas the reinforced composites presented two distinct exothermic peaks, reflecting contributions from both the polymer matrix and SiC filler.

Notably, the 0.2% nanofiber composite exhibited the second-highest heat flow after the 0.3% hybrid, suggesting greater crystallinity compared to the control, which displayed the most amorphous character and lowest heat flow.

## Conclusions

Nano-fiber and nano-hybrid dental composites were developed and evaluated in order to examine the impact of SiC incorporation on their mechanical, tribological, and morphological properties. The following is a summary of the main findings:

- When compared to the control group, the nanofiber-reinforced and nanohybrid composites showed improved hardness.
- The compressive strength increased when SiC nanofibers were used alone instead of hybrid reinforcement.
- The structural change of the dental restorative material from an amorphous to a semi-crystalline phase is facilitated by SiC nanofibers.
- Analysis using scanning electron microscopy (SEM) showed that the 0.2 wt% nanofiber composite and the 0.3 wt% nanohybrid composite had better surface morphology.
- The nanohybrid composite showed improved thermal stability and a more ordered structure according to differential scanning calorimetry (DSC).

### Limitations and future work

Despite the promising mechanical, tribological, and thermal performance of the developed composites, this study has some limitations. First, biological assessments, including cytocompatibility and long-term biocompatibility, were not performed. Second, corrosion resistance and wear behavior under saliva-like or wet conditions were not fully evaluated, which are critical for dental implant applications. Future work will address these aspects by conducting comprehensive in vitro biological testing, electrochemical corrosion studies, and lubricated wear experiments, as well as exploring additional filler types and concentrations to enhance the material’s clinical relevance.

## Data Availability

The dataset used and analysed during the current study is available from the corresponding author on reasonable request.
